# The Lords' Committee

**Published:** 1891-03-07

**Authors:** 


### The Lords' Committee.

Lord Sandhurst was in the chair last week, when the Westminster Hospital management was enquired into. Mr. S. Quennel, the Secretary, gave evidence. The hospital was practically free, the income was close on £40,000. Practically the patients were admitted by the senior house physician and surgeon, and they were admitted at all hours of the day and night. Small-pox, scarlet fever, and typhus fever cases were excluded. Diphtheritic cases were admitted, but they were not isolated as a general practice. Isolation depended upon the severity of the cases. The only restriction as to the number of out-patients admitted was that the physicians were not obliged to see more than 20 new cases. Last year 2,730 in-patients were received, and 27,026 out-patients were treated. Those were all new cases. On the whole he did not think the charity was abused. He considered that the medical relief in the district was sufficient for the requirements. The cost per bed last year was £62 18s. 4d. ; the year before £66 5s. 6d. ; and the year before that £62 18s. 9d. The witness said he assumed that he was responsible for the sanitary condition of the hospital. There was, however, an architect. There would, he thought, be some difficulty in an outside body inspecting the drains annually to see if they were in a satisfactory state. Asked as to what difficulty there could be in an experienced sanitary engineer giving an opinion on the subject, the witness replied that as they had their own architect it would cause some inconvenience if anyone else were to disturb the drains. In reply to Lord Cathcart, the witness said that there was a drain plan of the hospital kept up to date. Miss M. J. Pyne, the Matron of the hospital, next gave evidence as to the nursing department of the charity. She said that no difficulty arose from the fact that the hospital was served by nurses coming from a separate institution. The institution was started in 1874. There were, she said, a total of eight ward sisters, thirty-two hospital nurses, and twenty-three probationers. There were no paying probationers. From a large private nursing department connected with the institution, a number of nurses were sent out to private cases, the charge for which was from one and a-half to two guineas per week. She did not consider the nurses were overworked at the hospital. Dr. Allchin, the Dean of the medical school at the hospital, stated that he was also a lecturer at the hospital, and had medical charge of the nurses. There were 100 students. The average receipts of the school were £1,860, and the expenses £913. The balance was divided among the staff and lecturers. The maximum received by any one of the staff was not much over £100. He thought that infirmaries might be much more utilised by students for educational purposes. There was no ground for complaint in not having enough subjects, although it might be desirable to have a few more. All the physicians and surgeons held the London diploma. They had to by the laws of the hospital. He did not think the hospital lost any good men by such a rule. He was strongly adverse to special hospitals.

On Monday last Mr. Nixon, Secretary to University College Hospital, gave evidence. They had 207 beds. There was a large medical school and a large out-patients' department. There were only two dispensaries to his knowledge in the district. They sometimes were compelled to send away patients because of an insufficient number of beds. Last year they treated 41,732 new cases. It was impossible to keep the attendances; but he thought they averaged about 3½ to each case. The income of the hospital last year was £19,334, and the expenditure £19,560, leaving a deficit of £226. The income consisted chiefly of £2,020 from annual subscriptions, of £2,947 from dividends, £1,973 from legacies, and £7,853 from donations. They had a permanent endowment of £112,042. The nursing work of the hospital was done by a Church of England sisterhood; but Roman Catholics were admitted. This system of nursing was found to answer very well. Sister Cecilia (Sister Superior of All Saints' Home) stated that there were, besides herself, fifty-six nurses and thirteen probationers. The hospital paid a stated sum annually, and the sisterhood in return was obliged to provide sufficient nurses. The nurses had a month's holiday every year, and although the work was very trying, she did not think them over-worked. Dr. Barlow said he had been connected with the hospital for ten years. He was associated with the out patient department, which, although the hospital was not perfect, was not over-crowded. He considered it advisable to have an inquiry officer attached to that department of all hospitals. He was satisfied with the nursing arrangements. It was desirable to have a new hospital with an aërial zone around it. He did not think the poor suffered in any way from the presence of students in the wards. In fact, hospitals with medical schools attached were more popular among the poor than those which had no school. Mr. Barker, F.R.C.S., a surgeon at the hospital since 1875, having been examined, Mr. Berkeley Hill (late Dean of the medical school attached to the hospital) gave some statistics with regard to the institution. The school expenses were paid by the Council, and did not come out of the funds of the charity. He thought there were enough medical teachers in London, but some of the schools were not properly equipped. Mr. Brudenell Carter, F.R.C.S., examined by the Chairman, gave it as his very strong opinion that the effect of special hospitals was, to a great extent, to deprive the general hospitals of means of teaching, which were greatly required for the proper instruction of the students. He thought that the treatment at general hospitals was quite as good as that at special hospitals. He thought that now the general hospitals had separate departments for special diseases the functions of the special hospitals had passed away.

